# Co-Morbidities of Interstitial Cystitis

**DOI:** 10.3389/fnins.2012.00114

**Published:** 2012-08-10

**Authors:** Gisela Chelimsky, Elizabeth Heller, C. A. Tony Buffington, Raymond Rackley, Di Zhang, Thomas Chelimsky

**Affiliations:** ^1^Department of Pediatric Gastroenterology, Medical College of WisconsinMilwaukee, WI, USA; ^2^Johns Hopkins UniversityBaltimore, MD, USA; ^3^Veterinary Clinical Sciences, The Ohio State UniversityColumbus, OH, USA; ^4^Department of Urology, The Cleveland Clinic FoundationCleveland, OH, USA; ^5^Department of Neurology, Case Western Reserve UniversityCleveland, OH, USA; ^6^Department of Neurology, Medical College of WisconsinMilwaukee, WI, USA

**Keywords:** IC/BPS, co-morbidities, migraine headache, orthostatic intolerance, functional gastrointestinal disorders

## Abstract

**Introduction:** This study aimed to estimate the proportion of patients with interstitial cystitis/painful bladder syndrome (IC/BPS) with systemic dysfunction associated co-morbidities such as irritable bowel syndrome (IBS) and fibromyalgia (FM). **Materials and Methods:** Two groups of subjects with IC/BPS were included: (1) physician diagnosed patients with IC/BPS and (2) subjects meeting NIDDK IC/PBS criteria based on a questionnaire (ODYSA). These groups were compared to healthy controls matched for age and socio-economic status. NIDDK criteria required: pain with bladder filling that improves with emptying, urinary urgency due to discomfort or pain, polyuria >11 times/24 h, and nocturia >2 times/night. The ODYSA instrument evaluates symptoms pertaining to a range of disorders including chronic fatigue, orthostatic intolerance, syncope, IBS, dyspepsia, cyclic vomiting syndrome, headaches and migraines, sleep, Raynaud’s syndrome, and chronic aches and pains. **Results:** IC/BPS was diagnosed in 26 subjects (mean age 47 ± 16 years, 92% females), 58 had symptoms of IC/BPS by NIDDK criteria (mean age 40 ± 17 years, 79% females) and 48 were healthy controls (mean age 31 ± 14 years, mean age 77%). Co-morbid complaints in the IC/BPS groups included gastrointestinal symptoms suggestive of IBS and dyspepsia, sleep abnormalities with delayed onset of sleep, feeling poorly refreshed in the morning, waking up before needed, snoring, severe chronic fatigue and chronic generalized pain, migraines, and syncope. **Discussion:** Patients with IC/BPS had co-morbid central and autonomic nervous system disorders. Our findings mirror those of others in regard to IBS, symptoms suggestive of FM, chronic pain, and migraine. High rates of syncope and functional dyspepsia found in the IC/BPS groups merit further study to determine if IC/BPS is part of a diffuse disorder of central, autonomic, and sensory processing affecting multiple organs outside the bladder.

## Introduction

Interstitial cystitis/painful bladder syndrome (IC/BPS) is a syndrome characterized by urinary urgency, frequency, nocturia, and pain in the pelvis that worsens as the bladder fills and improves with emptying (Clemens et al., 2005; Bogart et al., 2007). IC/BPS may affect from 0.5 to 12% of women in the US (Jones and Nyberg, 1997; Clemens et al., 2005) and the quality of life for patients with interstitial cystitis is significantly degraded (Buffington, 2004). IC/BPS associates with other disorders such as irritable bowel syndrome (IBS), Sjogren’s syndrome, fibromyalgia (FM) syndrome, chronic fatigue syndrome, anxiety disorders, migraines, and other pain syndromes not related to the bladder. Moreover, these other conditions frequently precede the onset of bladder symptoms (Rodriguez et al., 2009; Warren et al., 2009; Hanno et al., 2010; Nickel et al., 2010). Interestingly, functional gastrointestinal disorders (FGID) like IBS, which often accompanies IC/BPS, have similar co-morbid symptoms as IC/BPS (Chelimsky et al., 2012). Furthermore, IBS harbors co-morbid dysautonomias similar to chronic fatigue (Wyller et al., 2010; Okamoto et al., 2011), FM (Reyes del Paso et al., 2011), and migraine headaches (Rashed et al., 1999). Although still unclear, a common theme to this group of co-morbid disorders could be related to the autonomic nervous system which connects the nervous system to the end-organ. Given the frequent co-existence of these disorders, the aim of this study was to evaluate if a similar number of additional co-morbid diagnoses that are present in FGID may also be associated with IC/BPS and contribute to the poor quality of life.

## Materials and Methods

This cross-sectional IRB-approved review used the Ohio Dysautonomia (ODYSA) questionnaire, a thorough clinical instrument designed to approximate the diagnosis of several syndromes that may have associated autonomic dysregulation including: orthostatic intolerance, reflex syncope, cyclic vomiting syndrome (CVS), interstitial cystitis, Raynaud’s syndrome, complex regional pain syndrome (CRPS), IBS, functional dyspepsia, functional abdominal pain, migraine headache, FM, and chronic fatigue syndrome. Where validated, published question-based diagnostic were utilized directly or slightly modified (Melzack, 1987; Fukuda et al., 1994; Merskey and Bogduk, 1994; Chelimsky et al., 1995; Drossman et al., 2000; Sheldon et al., 2002, 2006; Olesen, 2004; Li et al., 2008; Low and Benarroch, 2008).

All patients who came to the Autonomic Laboratory at University Hospital Case Medical Center for testing for any type of complaint are asked to complete the ODYSA questionnaire as part of their clinical care, as well as patients seen in urology, neurology, rheumatology, and gastroenterology interdisciplinary autonomic clinics. There were no exclusion criteria.

Control subjects constituted a participant’s same gender friend or the spouse’s same gender sibling, with intent to closely match socio-economic and geographic factors. The ODYSA questionnaire includes the O’Leary–Sant question-set (O’Leary and Sant, 1997) as well as a separate face-valid question-set designed to assess the probability that IC/BPS is present based on most recent NIDDK (2006) criteria. The probability of having the disease is forced to 0 or 1 (no intermediate values), and was programmed into a database (Filemaker) to automatically generate a score for each subject as the data are entered, obviating any opportunity for subjective interpretation. Data entry was performed by students who had no knowledge of patient diagnosis and was double-checked. NIDDK criteria for IC/BPS were as follows: (1) the subject’s pain must worsen with a full bladder and improve with an empty bladder; (2) urinary urgency must result from discomfort or pain (not fear of incontinence); (3) and voiding frequency must exceed 11 times on average in a 24 h period, including at least twice per night. Each criterion scored 1 if fulfilled and 0 otherwise with a total possible range of 0–5, and 4 or greater considered likely IC/BPS.

Three subject groups were used in this study. The first group included those subjects with a clinical diagnosis (ICDx) of IC/BPS made by a specialty physician, a urologist, uro-gynecologist, or gynecologist. The second group comprised subjects with symptoms of IC/BPS based on the NIDDK criteria as per ODYSA question-set (ICSx), and the third group included control subjects enrolled (spouse or friend) who did not meet criteria for IC/BPS. The exclusion criteria were only established for the control subjects, in that they could not meet criteria for IC/BPS. We examined each group for co-morbid disorders using the following criteria based on question-set answers. The symptoms were suggestive of IBS (periumbilical/lower abdomen abdominal discomfort for >6 months with changes in bowel movement frequency or consistency or relieved by a bowel movement) or for dyspepsia (discomfort in chest or upper abdomen with one of the following: bloating, early satiety, or nausea). For symptoms suggestive of CVS, the subjects needed to report more than five episodes in their lifetime of severe stereotypical episodes of nausea or vomiting with return to baseline health in-between. Dizziness was defined by reporting one or more of the following symptoms “when you stand still or exercise a little…” one time per day to two times per week: feel faint, dizzy, lightheaded, or noticed change in vision or thinking is “off.” For the history of syncope, the question was phrased as “Do you ever faint (completely lose consciousness)?” We considered positive if >three times/lifetime. Several questions were asked about sleep issues: “Does it take more than half an hour to fall asleep?” (considered delayed onset of sleep if >30 min), “Do you snore or stop breathing when you sleep?,” “Do you wake up before you need to?” “Do you force yourself to stay awake during the day?” (daytime sleepiness), “Do you feel refreshed after you sleep?” For the fatigue, it was phrased as “Do you have unexplained severe fatigue lasting…,” being considered positive if >6 months. To have significant headaches, the subjects had to report headaches >50/lifetime and complain of a throbbing quality and moderate to severe intensity (migrainous features). To assess for possible Raynaud’s syndrome, we asked if the fingers turned white and turned red in cold temperatures and became painful.

Statistical methods were performed utilizing Microsoft Excel 2010. The Chi-square test was utilized to determine if the individual co-morbid disorders were different between the two IC/BPS groups and between each of the IC/BPS groups and the control group. Statistical significance was considered for *p* < 0.05.

## Results

**Figure 1:**
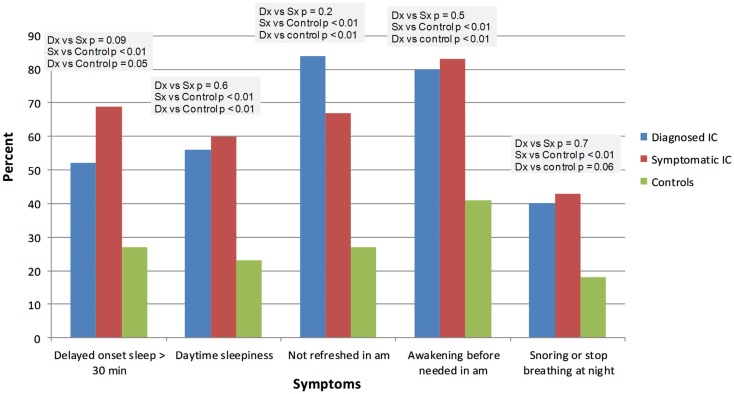
**Comparison of effects of IC/BPS on sleep function between healthy controls and patients diagnosed by physician or questionnaire**. Dx: corresponds to IC/BPS diagnosed by a physician (diagnosed IC); Sx corresponds to subjects who have symptoms of IC/BPS based on NIDDK criteria (symptomatic IC).

**Figure 2:**
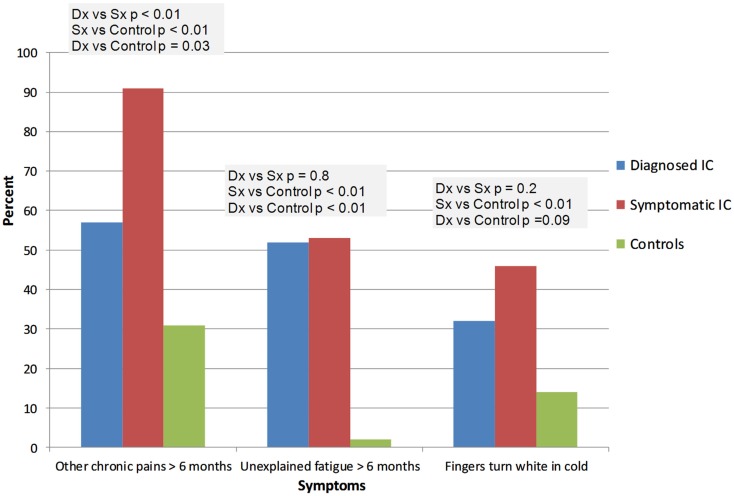
**This figure summarizes the complaints of fatigue, chronic pains, and Raynaud’s like symptoms**. Dx: corresponds to IC/BPS diagnosed by a physician (diagnosed IC); Sx corresponds to subjects who have symptoms of IC/BPS based on NIDDK criteria (symptomatic IC). “Fingers turning white in cold” is purposed for looking at signs of possible Raynaud’s syndrome.

**Figure 3:**
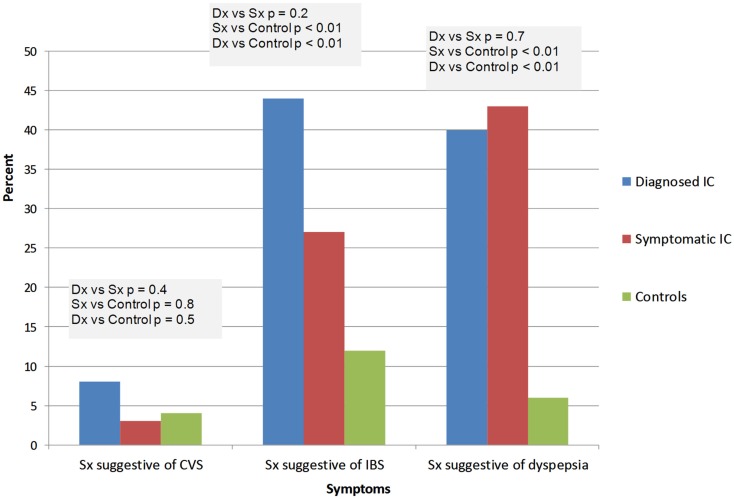
**Summary of the gastrointestinal complaints**. Dx: corresponds to IC/BPS diagnosed by a physician (diagnosed IC); Sx corresponds to subjects who have symptoms of IC/BPS based on NIDDK criteria (symptomatic IC). CVS, cyclic vomiting syndrome; IBS, irritable bowel syndrome.

**Figure 4:**
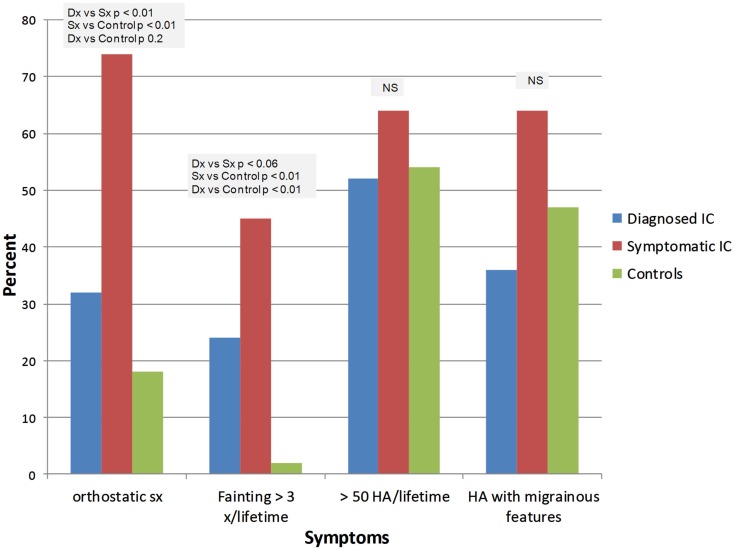
**Summary of orthostatic symptoms and headaches**. Dx: corresponds to IC/BPS diagnosed by a physician (diagnosed IC); Sx corresponds to subjects who have symptoms of IC/BPS based on NIDDK criteria (symptomatic IC).

IC/BPS was diagnosed by a physician in 26 subjects (ICDx group: mean age 47 ± 16 years, 92% females), while 58 subjects had symptoms of IC/BPS according to NIDDK IC/BPS criteria (ICSx group mean age 40 ± 17 years, 79% females), and 48 were healthy controls (mean age 31 ± 14 years, 77% females). The most common co-morbid complaints in both IC/BPS groups included gastrointestinal symptoms, sleep abnormalities, severe fatigue and chronic pain, headache, and syncope (Figures 1–4). Orthostatic symptoms were reported in 74% of ICSx subjects and in 32% of ICDx. Syncope was also more prevalent in the ICSx group (45%) than in ICDx (24%). This difference in orthostatic symptoms and syncope probably reflects a referral bias, since subjects are frequently referred to the autonomic laboratory for orthostatic complaints. The other symptoms were present in the two IC/BPS groups without significant difference. In relation to sleep, both groups reported taking >30 min to fall asleep (ICDx = 52%; ICSx = 69%), daytime sleepiness (ICDx = 56%, ICSx = 60%), not feeling refreshed in the morning (ICDx = 84%; ICSx = 67%), awakening before needed in the morning (ICDx = 80%; ICSx = 83%), and snoring or stopping breathing at night (ICDx = 40%; ICSx = 43%). In relation to gastrointestinal symptoms, cyclic vomiting like symptoms were uncommon in all IC/BPS groups, but symptoms suggestive of IBS (ICDx = 44%; ICSx = 27%; controls = 12%) and dyspepsia (ICDx = 40%; ICSx = 43%; controls = 6%) were significantly more common in IC/BPS subjects. IC/BPS subjects had also significantly more complaints of chronic pains lasting longer than 6 months (ICDx = 57%; ICSx = 91%; controls = 31%) and fatigue lasting longer than 6 months (ICDx = 53%; ICSx = 53%; controls = 2%). Interestingly, we could not find a difference between the two groups and the controls in prevalence of migraine headaches though power was too low to know if a difference was truly absent.

## Discussion

The present study adds to the list of co-morbid symptom complexes in subjects with IC/BPS, including dyspepsia-like symptoms, chronic body-wide pains, and orthostatic complaints. Our findings support the previously described associations of migraine headaches, FM, IBS, CRPS, significant sleep abnormalities, and chronic fatigue (Nickel et al., 2009, 2010; Tsai et al., 2009; Warren et al., 2009). These co-morbid symptom complexes probably contribute to poor quality of life in ways that physicians may overlook, particularly in a uni-disciplinary context. Importantly, they also influence our conceptualization of this syndrome as originating from some type of systemic process, or from a central nervous system derangement, rather than from the end-organ.

Much of the literature assessing quality of life finds a strong positive correlation between quality of life and marital status, sexual function, and employment (Nickel et al., 2007). These co-morbidities likely contribute to poor quality of life, though patients themselves may not mention them unless specifically queried. For this reason, many centers, including ours, now utilize an interdisciplinary clinic with urology-gynecology, neurology, psychology, anesthesia/pain management, and rehabilitation services. Approaches that address sleep, mood, and chronic pain, for example through the use of a tricyclic agent, and that stress physical reconditioning through exercise may have specific salutary effects on the entire syndrome, not just the end-organ that constitutes the particular focus of the patient’s complaint.

The high frequency of co-morbid disorders well beyond geographic contiguity with the bladder region (e.g., headache, sleep disorders, fatigue, etc.) affects our pathophysiologic construct of IC/BPS, suggesting either some type of systemic disorder, a process under central nervous system control, or both. The absence of any frank neuro-inflammation (Nickel, 2002) reduces the likelihood of a disorder of cellular immunity or cytokine activation. An abnormal central nervous system drive, with secondary psycho-neuro-endocrine-immune dysfunction (Irwin and Cole, 2011) seems likely, as occurs in FM, where heart rate variability analysis demonstrates a skew toward low sympathetic frequencies with reduction or even absence of high parasympathetic frequencies (Staud, 2008). In migraine, an autonomic neuropathy occurs frequently (Rashed et al., 1999) with a similar skew favoring sympathetic over parasympathetic activity.

Although no good data yet exist in patients with IC/BPS, studies in the co-morbid disorder FM have identified several areas of the brain that are activated with application of pain that are not activated in healthy individuals. This “pain matrix” is currently conceptualized as reflecting an afferent processing disorder (Smith et al., 2011). Since the primary brain abnormality must involve both afferent pain processing and efferent autonomic processing simultaneously, good candidates include brainstem structures such as the locus ceruleus or the raphe nuclei. The raphe are particularly attractive as a hypothetical region of origination since they are deeply involved in sleep regulation (Monti, 2010) as well as in the probable generation of migraine headache and associated phenomena (Pringsheim et al., 2003), one of which occurs in many if not the majority of patients with most functional disorders. The raphe are also in close proximity to control areas involved in continence, which include the pons, the periaqueductal gray, the thalamus, insula, anterior cingulate, and prefrontal cortex (Kavia et al., 2005).

The patient group diagnosed with IC/BPS (ICDx) was remarkably similar to the group identified by questionnaire (ICSx) and both differed from control subjects. According to the IC Database study, NIDDK criteria used for ICSx are more restrictive than specialist opinion (ICDx; Hanno et al., 1999), and this could explain the higher rate of “other pains,” if they represent a slightly more severe subgroup. The higher rate of orthostatic disorders in the ICSx group represents a referral diagnosis bias to the autonomic lab and clinic from where this population was drawn. The rate of migraine in the healthy control group of 50% was higher than a recent Norwegian study which found a migraine prevalence of 35% (Vetvik et al., 2010), perhaps due to a young predominantly female population that matched the experimental groups in gender, age, and socio-economic status. However, other common functional disorders like IBS, dyspepsia, aches, and pain, were not increased in the control population.

This study has several limitations. Whereas the ICDx group was diagnosed objectively by a physician specializing in interstitial cystitis, the ICSx group used a patient survey with its attendant errors, subject recall-bias, questionnaire fatigue, and misunderstandings of questions. The highly similar occurrence of co-morbid disorders in both groups is reassuring, as is the fact that many ICDx subjects did not meet NIDDK criteria on the questionnaire, since an identical finding occurred with patients in the IC/BPS Database study (Hanno et al., 1999). Finally, moderate patient numbers could lead to a type II error, finding no difference between groups when a difference actually exists. A moderate sample size is not likely to suggest a difference when the truth is that the groups are identical.

In conclusion, this study demonstrates widespread co-morbidities in patients with interstitial cystitis, both physician and questionnaire diagnosed, with very similar findings in the two groups. Known co-morbid disorders were confirmed, including migraine headache, IBS, and widespread pain. New co-morbidities emerged, including dyspepsia-like symptoms and orthostatic intolerance. The multiple involvement of organ systems far from the bladder supports the theory that IC/BPS is not a primary bladder disorder (Warren et al., 2011), but rather the bladder is one more organ system involved in a systemic, possibly neurologic disorder.

## Conflict of Interest Statement

The authors declare that the research was conducted in the absence of any commercial or financial relationships that could be construed as a potential conflict of interest.
